# Endocytosis and Nephrotoxicity—It's a RAP!

**DOI:** 10.34067/KID.0000000000000144

**Published:** 2023-05-25

**Authors:** Linda Awdishu, Melanie S. Joy

**Affiliations:** 1 Division Head of Clinical Pharmacy, University of California, San Diego Skaggs School of Pharmacy and Pharmaceutical Sciences; 2 Department of Pharmaceutical Sciences, Director, Pharmaceutical Science Innovation and Commercialization, University of Colorado, Skaggs School of Pharmacy and Pharmaceutical Sciences; **Correspondence:** Dr. Linda Awdishu, Division of Clinical Pharmacy University of California, San Diego Skaggs School of Pharmacy and Pharmaceutical Sciences, 9255 Pharmacy Lane, MC 0657, La Jolla, CA 92093-0657. Email: lawdishu@health.ucsd.edu

**Keywords:** AKI, cell and transport physiology, drug nephrotoxicity, endocytosis, gentamicin

**Figure 1:**
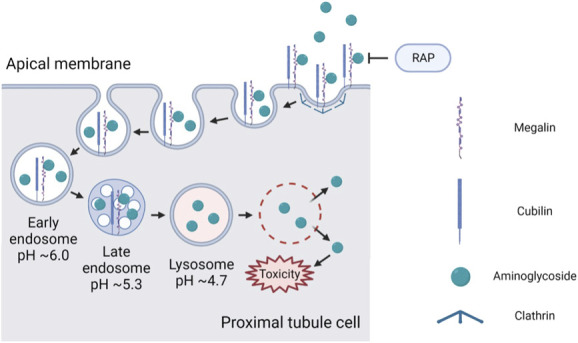
**The proposed role of RAP in endocytosis and mitigation of gentamicin nephrotoxicity.** This figure depicts the role of RAP as an inhibitor of clathrin-mediated endocytosis. Gentamicin binds to megalin and cubulin on the apical membrane of proximal tubule cells and is invaginated in clathrin-coated pits, followed by budding and fission. Gentamicin enters early endosomes, followed by trafficking to late endosomes and lysosomes under acidification conditions. Lysosomes degrade causing toxicity to proximal tubule cells. RAP, receptor-associated protein. Figure made using BioRender.com and adapted from Stöppler D, Macpherson A, Smith-Penzel S, *et al.* Insight into small molecule binding to the neonatal Fc receptor by X-ray crystallography and 100 kHz magic-angle-spinning NMR. *PLoS Biol*. 2018; 16(5):e2006192. doi:10.1371/journal.pbio.2006192

Wagner *et al.* report interesting findings that implicate *α*-2 macroglobulin receptor–associated protein, Lrpap1 (RAP), not only in the expected inhibition of clathrin-mediated endocytosis but also in the inhibition of clathrin-independent (fluid phase) endocytosis in the renal proximal tubule (PT) implicating a role for megalin in fluid phase endocytosis.^1^ They also provide evidence of some protection from aminoglycoside nephrotoxicity through inhibition of PT endocytosis of gentamicin when stable RAP (sRAP; i.v. 40 mg/kg) was exogenously administered to Munich Wistar Fromter (MWF) rats with chronic kidney disease (Figure 1).

Receptor-mediated endocytosis involves interaction of a ligand with membrane receptors and adaptor proteins and invagination in clathrin-coated pits, followed by budding and fission.^2^ Internalized proteins enter into early endosomes, followed by trafficking to late endosomes and lysosomes where they undergo degradation and/or transport.^3^ The progression along the endocytic apparatus to lysosomes depends on endosomal acidification, which is achieved by H^+^-ATPase coupled to chloride conductance.^4^ Megalin (low-density lipoprotein receptor-related protein) and cubilin are recognized for their involvement in receptor-mediated endocytosis, and they associate with adaptor proteins for their normal function.^3^ Megalin is a well-known 600 kDa transmembrane protein from the low-density lipoprotein receptor family which binds a variety of ligands including albumin, hemoglobin, vitamin carrier proteins, lipoproteins, hormones, enzymes, and aminoglycosides.^5^ Cubilin, a 460 kDa extracellular protein, forms a complex with megalin, leading to internalization with bound ligands. The extracellular motifs and ligand binding sites of megalin are identical in humans and rats.^6^ RAP is a chaperone protein to megalin which when administered exogenously is a potent antagonist inhibiting binding of ligands to megalin and cubilin.^2,7^ Megalin interaction with RAP leads to folding and biosynthetic trafficking of megalin and accompanies megalin to the plasma membrane and dissociates from the receptor.^2^ The chloride channel-5 expressed in kidney PT cells is localized in endosomes mediating endosomal acidification and recycling of megalin to the cell membrane.^4^ Gentamicin is freely filtered by the glomerulus, and because of its positive charge, it binds to megalin on the apical side of proximal tubular cells for receptor-mediated endocytosis into the cell and subsequent accumulation in lysosomes.^8^ After entry into PT cells, gentamicin can induce intracellular toxicity *via* lysosomes, Golgi apparatus, and cytosol.

The purpose of the study was to characterize the PT handling of RAP and determine its effects on subsequent endocytosis. PT convoluted cells in the S1 and S2 segments are known to express high levels of megalin and cubilin on the apical membrane, which mediate the uptake of aminoglycosides and other nephrotoxins such as colistin and polymyxin B from the filtrate.^2^ The investigators used MWF rats (8–10 weeks) which have surface glomeruli to facilitate imaging, leveraging their expertise in intravital microscopy for the real-time visualization of glomerular filtration, PT cell uptake, and cell trafficking. A tracer dose (3 mg/kg) of Texas Red conjugate of Lrap1 was used to demonstrate RAP binding to the apical brush border, early uptake, and distribution throughout the PT cells primarily in the S1 segment.

Following this study, Texas Red-X rat serum albumin conjugate and Cascade Blue anionic dextran were used to assess clathrin-mediated and clathrin-independent PT endocytosis, respectively. Nonfluorescent RAP at a dose of 40 mg/kg was used to study the inhibition of endocytosis pathways and was administered before the fluorescent endocytosis markers anionic dextran and rat serum albumin. Acute assessment of endocytosis inhibition was performed at 60 minutes while recovery of function was evaluated after 4 hours. Administration of RAP before Cascade Blue anionic-dextran resulted in a 6-fold reduction in uptake, predominantly in the S1 segment of the PT. Administration of RAP before Texas Red-X rat serum albumin conjugate resulted in a 2–2.5-fold reduction in albumin uptake, predominantly in the S1 segment of the PT. Reversal studies showed increased time to recovery for PTs downstream of S1, with only partial recovery at 5 hours. These results suggest that RAP is a potent inhibitor of clathrin-dependent and clathrin-independent endocytosis pathways. However, additional studies are needed to fully evaluate the optimal RAP dose and the time course effects on inhibition and full recovery of these pathways to assess the true feasibility of this approach toward clinical translation. Justification of the selected RAP dose for inhibition of 40 mg/kg was not provided. Exogenously administered RAP could bind to other sites containing megalin such as cochlear cells and could also inhibit binding of other ligands such as vitamin D in PT cells, necessitating further investigation into off-target consequences and to determine whether RAP inhibition of endocytosis causes detrimental effects.

A proof-of-concept evaluation was also conducted and demonstrated that RAP and sRAP administration reduced gentamicin (megalin substrate) uptake by 2-fold, with sRAP demonstrating greater inhibition and partial reversal at 5 hours. The investigators then demonstrated that sRAP mitigates gentamicin nephrotoxicity when administered 100 mg/kg i.p. for 5 days in a CKD MWF model.^9^ The sRAP-treated rats exhibited an attenuation but not absence of elevations in serum creatinine and urine protein excretion and reductions in glomerular filtration rate as compared with rats that were not treated with sRAP. The lack of a full response could be due to inadequate sRAP dosing, timing of administration, and/or other noninhibited pathways for gentamicin entry into PT cells. The histological evaluations, however, were not overly remarkable. Full pharmacokinetic and pharmacodynamic evaluations will be required to identify optimal dosing for the intended duration of endocytosis inhibition after administration of a known nephrotoxin to prevent kidney injury. Given the antagonism of endocytosis, it will be prudent to evaluate the effect on relevant proteins, peptides, and vitamins (including vitamin D) which use endocytosis for maintaining homeostasis.

There have been numerous approaches investigated to date that have sought to prevent gentamicin nephrotoxicity, including megalin ligands such as cytochrome C and cilastatin as an inhibitor of renal dehydropeptidase-I and competitive inhibitor of megalin binding.^10^ Studies of megalin ligands have been limited because of potency, and design of receptor antagonists on the basis of receptor structure could improve therapeutic efficacy. Other potential mitigation strategies have focused on reducing endosomal acidification using montelukast which reduces expression of chloride channel-5 and interferes with megalin recycling to the cell membrane.^11^ The net effect in reduced expression of megalin and reduced endocytosis of gentamicin.^11^

In conclusion, the current publication informs about the role of RAP inhibition of clathrin-mediated and clathrin-independent (fluid phase) endocytosis in the renal PT. The evidence of inhibition of PT endocytosis of gentamicin and partial mitigation of kidney injury with sRAP is encouraging as a potential therapeutic strategy warranting further investigation. Further work is needed to identify the optimal dosing and determine off-target effects. Insights into the underlying mechanisms of endocytosis of nephrotoxins will reveal new targets for inhibition of cell entry to alleviate nephrotoxicity and possibly other end-organ toxicities.
